# New multidisciplinary prostate bone metastases clinic: first of its kind in Canada

**DOI:** 10.3747/co.2007.101

**Published:** 2007-02

**Authors:** P. Goh, K. Harris, J. Napolskikh, E. Chow, E. Sinclair, U. Emmenegger, S. Lemon, A. Yee, L. Wynnychuk, J. Myers, C. Danjoux, Y. Ko, (The Bone Metastases Site Group)

**Keywords:** Prostate, bone metastases, prostate clinic, skeletal-related events, hormone-refractory, multidisciplinary, Toronto–Sunnybrook Regional Cancer Centre, zoledronic acid, bisphosphonate

## Abstract

Prostate cancer is the most common non-skin malignancy in men. Almost all men who die from prostate cancer have hormone-refractory prostate cancer with metastasis to bone. Emerging supportive treatments—including chemotherapy, bisphosphonates, and surgery—require integration that is optimized in a multidisciplinary setting. A multidisciplinary clinic for bone metastases has been in place at Toronto–Sunnybrook Regional Cancer Centre since 1999, combining orthopedic surgery, radiation oncology, interventional radiology, and palliative medicine for all patients with bone metastases. The addition of a prostate-focused multidisciplinary clinic integrates these services for patients with advanced prostate cancer.

## 1. INTRODUCTION

Prostate cancer is the fourth most common cancer worldwide and the most common cancer in Canada among men 1,2. An estimated 20,500 new cases of prostate cancer (pca) occurred in 2005 in Canada, and an estimated 4300 men will die as a result 1. In Ontario alone, pca was found to be the most frequently diagnosed cancer, with an estimated 8300 new cases in 2005^1^. Most patients with advanced disease will develop metastases. The most common metastatic site is bone, with bone metastases being found in approximately 80% of advanced pca patients^3^.

Over recent decades, patient survival times have increased because of improved radiotherapy and systemic treatments 4,5. Analgesic therapy and radiation therapy to bone have been the cornerstone of palliation in patients with hormone-refractory pca meta-static to bone. The recent addition of taxane-based chemotherapy and bisphosphonates is helpful, but many patients may not be accessing these valuable therapies in a timely manner. The establishment of a prostate bone metastasis clinic in a tertiary care centre will provide timely multidisciplinary care to patients with advanced pca.

## 2. DISCUSSION

### 2.1 Metastatic Occurrence in Bone

Cancer metastasis to bone can occur in hormone-sensitive disease, but it may also develop or progress during the hormone-refractory stage. In prostate cancer, metastases tend to be more osteoblastic than osteolytic: cancer cells produce factors that stimulate osteoblast proliferation, causing increased abnormal bone formation rather than bone breakdown 6. Although androgen deprivation therapy is highly effective in treating hormone-sensitive pca, almost all patients become resistant after a median of 18–24 months. In the setting of castrate serum testosterone levels, resistance is often indicated by a rising level of prostate-specific antigen (psa) before symptoms progress. With continuing improvement in systemic therapies such as chemotherapy, the overall survival of patients with hormone-refractory pca is increasing^3^. However, recent studies have shown that androgen deprivation therapy has harmful effects on the skeleton. The accelerated bone loss potentially puts men at higher risk of skeletal complications 3.

### 2.2 Complications

Bone metastases are not life-threatening in themselves, but their presence generally indicates a poor prognosis^7^. In pca patients, the most common skeletal sites of metastases are the pelvic bones, vertebral column, ribs, and long bones, particularly the femur and humerus^7^,^8^. Predictors of skeletal morbidity include extensive bone lesions seen on diagnostic imaging, poor performance status, severe bone pain, and an increase in psa level^8^.

Morbidity stems from the various complications from bone metastases. Skeletal-related events (sres) include debilitating bone pain in up to 80% of patients with bone metastases, impaired mobility in 65%–75%, vertebral collapse or deformity in 18%, hypercalcemia in 10%–15%, spinal cord compression in 12% of pca patients, and pathologic fracture in 9%^9^–^12^. These complications may cause deformity, postural problems, and loss of motor and sensory function (neurologic impairment), leading to de) creased overall quality of life (qol 13. Management of bone metastases has changed from solely providing pain relief to improving qol and survival^14^.

### 2.3 Available Treatments

Current treatment options for pca patients with bone metastases include external beam radiotherapy, surgery, systemic therapy (bisphosphonates, hormonal therapy, and chemotherapy), and management with pain medications 5,15. The goals of these therapies are to reduce the incidence of sres, reduce bone pain and morbidity, and improve the patient’s mobility and overall qol^2^,^5^. Patients with metastatic disease are treated with palliative intent. Each of the available treatments depends on the patient’s underlying health and performance status, the location of the metastases, the treating physician, and the facilities available in the local area^15^.

Analgesic therapy and radiation remains the treatment of choice for localized metastasis-related bone pain^12^. Radiation is also effective in preventing impending fractures, reducing bone pain, and promoting healing in pathologic fractures^16^. About 80%–90% of patients treated with radiotherapy for bone metastases experienced pain alleviation^16^. Unfortunately, some of these patients eventually experience a recurrence in pain at the previously radiated skeletal site^5^.

Patients with painful lesions larger than 2.5 cm in diameter and with more than 50% of cortical bone destroyed in a weight-bearing bone are at high risk of experiencing a pathologic fracture and may need surgical intervention 7. Internal fixation or prophylactic orthopedic fixation will allow for better qol through pain relief, stability maintenance, mobility, and recovery of weight-bearing capacity 14,16,17. Surgery is usually reserved for patients with good performance status and a life expectancy of at least 6 months so that sufficient time and strength are available for recovery and rehabilitation 12,18,19.

General anesthesia in surgical intervention is not without risk, especially for patients in advanced stages of cancer 14. For those with vertebral metastasis, an alternative surgical approach is vertebroplasty, which uses injections of polymethylmethacrylate bone cement into the vertebral body to restore spinal stability and relieve pain 14.

The advantage of systemic therapy is the ability to deliver the treatment throughout the body 20. Two large randomized studies established that docetaxel-based chemotherapy can improve median survival in patients with metastatic hormone-refractory pca 21,22. In addition, taxane-based chemotherapy is more effective in palliating symptoms of metastatic pca than are mitoxantrone and prednisone 23. Also, bisphosphonates can help treat bone metastases in pca patients by inhibiting osteoclastic activity. However, several randomized studies of clodronate and pamidronate failed to demonstrate significant benefit in terms of pain relief 5,24,25. A randomized placebo-controlled study conducted by Saad *et al.*^26^,^27^ with a third-generation bisphosphonate—zoledronic acid—demonstrated a reduction in the number of sres and extension of the time to first sre by approximately 150 days. Based on that study, zoledronic acid has been approved by Health Canada and the U.S. Food and Drug Administration for the treatment of hormone-refractory pca.

Analgesic therapy requires a comprehensive pain assessment and an understanding of the causes of pain and of pain management. Opioids may then be appropriately initiated, titrated, and augmented with adjuvants including steroids, nonsteroidal anti-inflammatory agents, and a variety of other agents that specifically target the neuropathic pain commonly seen in the setting of bone metastases 28.

### 2.4 Poor Integration of Services

The variety of pca treatments has unfortunately translated into a lack of coordination between the specialists responsible for treating the patient. Treating bone pain caused by metastatic disease is a multifaceted task. At the moment, many patients wait for separate appointments to see each specialist (for example, the radiation oncologist, medical oncologist, orthopedic surgeon, and palliative medicine physician), often in sequence, which compounds wait times. Timing is key to the effective treatment of patients with impending pathologic fracture, spinal cord compression, and bone pain. Early diagnosis and treatment are vital for the patient’s qol and for the prevention of permanent or further neurologic damage 9,12.

### 2.5 New Multidisciplinary Prostate Bone Metastases Clinic

The current Bone Metastases Clinic (bmc) at Toronto–Sunnybrook Regional Cancer Centre has been operating since 1999. The bmc offers a multidisciplinary approach to the care of cancer patients with symptomatic bone metastases in a tertiary cancer center. The new multidisciplinary Prostate Bone Metastases Clinic (pbmc) will overlap with the bmc to help provide patients with the best possible care for their cancer.

The pbmc will be a one-stop clinic at which patients will have the opportunity to see a variety of doctors, based on need. The team of specialists will focus on providing the best therapeutic options and combinations, tailored specifically to the individual. The clinic will greatly facilitate direct communication between the health care specialists. Community urologists usually manage the care and make the referrals for pca patients receiving hormonal therapy. Therefore, pbmc will address the need for a simplified referral to an all-in-one clinic for patients requiring more than one specialist.

The pbmc will have a team of 5 radiation oncologists, 1 medical oncologist, 1 interventional radiologist, 3 orthopedic surgeons, 2 palliative medicine physicians, a radiation therapist, a primary nurse coordinator, and a research assistant. Each patient will have the opportunity to receive specialized care including radiation therapy, systemic therapy (chemotherapy, bisphosphonates, and hormonal therapy), pain management, surgical intervention, and supportive care where applicable. The patient will also benefit from the savings in time, money, and energy that would otherwise be spent on travel and waits for separate specialist appointments.

The new multidisciplinary pbmc will be held every Monday afternoon in conjunction with the regular bmc occurring on the second and fourth Friday of each month. Thereafter, the patients will be followed up at the medical oncologist’s regular clinic. The pbmc will provide optimal service for pca patients through its multidisciplinary approach. In addition, the venue will facilitate clinical research, such as novel bone-targeted therapies, chemotherapies, and improvements in radiation therapy, thereby augmenting the clinic’s ability to effectively treat patients with the latest research and evidence-based care.

## References

[1] National Cancer Institute of Canada. *Canadian Cancer Statistics 2005.* Toronto: National Cancer Institute of Canada; 2005.

[2] Saad F, Karakiewicz P, Perrotte P (2005). The role of bisphosphonates in hormone-refractory prostate cancer. World J Urol.

[3] Coxon JP, Oades GM, Colston KW, Kirby RS (2004). Advances in the use of bisphosphonates in the prostate cancer setting. Prostate Cancer Prostatic Dis.

[4] Coleman R, Neville–Webbe H. Prevention of bone metastases. In: Jasmin C, Coleman R, Coia L, Capanna R, Saillant G, eds. *Textbook of Bone Metastases.* Mississauga, ON: Wiley; 2005: 387–96.

[5] Brown J, Neville–Webbe H, Coleman R (2004). The role of bisphosphonates in breast and prostate cancers. Endocr Relat Cancer.

[6] Rabbani S, Shukeir N, Mazar A. Prostate cancer: models for developing novel therapeutic approaches. In: Singh G, Orr W, eds. *Bone Metastasis and Molecular Mechanisms Pathophysiology* Dordrecht: Kluwer Academic Publishers; 2004: 163–86.

[7] Hoskin P, Makin W. *Oncology for Palliative Medicine* New York: Oxford University Press; 2003: 271–89.

[8] Heidenreich A (2003). Bisphosphonates in the management of meta-static prostate cancer. Oncology.

[9] Body JJ (1992). Metastatic bone disease: clinical and therapeutic aspects. Bone.

[10] Bilezikian J (1992). Management of acute hypercalcemia. N Engl J Med.

[11] Orr F, Kostenuik P, Sanchez–Sweatman O, Singh G (1993). Mechanisms involved in the metastasis of cancer to bone. Breast Cancer Res Treat.

[12] Tazi H, Manunta A, Rodriguez A (2003). Spinal cord compression in metastatic prostate cancer. Eur Urol.

[13] MacKenzie M, Major P. The role of bisphosphonates in bone metastasis. In: Singh G, Orr W, eds. *Bone Metastasis and Molecular Mechanisms Pathophysiology* Dordrecht: Kluwer Academic Publishers; 2004: 277–301.

[14] McDuffee L, Colterjohn N, Singh G. Bone metastasis and pathological fractures: bone tissue engineering as a novel therapy. In: Singh G, Rubbani S, eds. *Bone Metastasis: Experimental and Clinical Therapeutics* Totowa, NJ: Humana Press; 2005: 229–41.

[15] Konski A (2004). Radiotherapy is a cost-effective palliative treatment for patients with bone metastasis for prostate cancer. Int J Radiat Oncol Biol Phys.

[16] Chow E, Wu J, Barnes E. Radiation treatment of bone metastases. In: Singh G, Rubbani S, eds*. Bone Metastasis: Experimental and Clinical Therapeutics* Totowa, NJ: Humana Press; 2005: 323–36.

[17] Hoskin P, Makin W. *Oncology for Palliative Medicine.* New York: Oxford University Press; 2003: 31–6.

[18] Coleman R. Use in prostate cancer. In: Jasmin C, Coleman R, Coia L, Capanna R, Saillant G, eds. *Textbook of Bone Metastases* Mississauga, ON: Wiley; 2005: 377–84.

[19] Fusi A, Procopio G, Della Torre S (2004). Treatment options in hormone-refractory metastatic prostate carcinoma. Tumori.

[20] Hoskin P, Makin W. *Oncology for Palliative Medicine* New York: Oxford University Press; 2003: 51–65.

[21] Joly F, Tannock I (2004). Chemotherapy for patients with hormone-refractory prostate cancer. Ann Oncol.

[22] Tannock I, Wit R, Berry W (2004). Docetaxel plus prednisone or mitoxantrone plus prednisone for advanced prostate cancer. N Engl J Med.

[23] Petrylak D, Tangen C, Hussain M (2004). Docetaxel and estramustine compared with mitoxantrone and prednisone for advanced refractory prostate cancer. N Engl J Med.

[24] Ernst D, Tannock I, Winquist E (2003). Randomized, double-blind, controlled trial of mitoxantrone/prednisone and clodronate versus mitoxantrone/prednisone and placebo in patients with hormone-refractory prostate cancer and pain. J Clin Oncol.

[25] Small EJ, Smith MR, Seaman JJ, Petrone S, Kowalski MO (2003). Combined analysis of two multicenter, randomized, placebo-controlled studies of pamidronate disodium for the palliation of bone pain in men with prostate cancer. J Clin Oncol.

[26] Saad F, Lipton A (2005). Zoledronic acid is effective in preventing and delaying skeletal events in patients with bone metastases secondary to genitourinary cancers. BJU Int.

[27] Saad F, Gleason D, Murray R (2002). A randomized, placebo-controlled trial of zoledronic acid in patients with hormone-refractory metastatic prostate carcinoma. J Natl Cancer Inst.

[28] Taylor G, Kurent J. *A Clinician’s Guide to Palliative Care.* Boston, MA: Blackwell Science Publishing Inc.; 2003.

